# Unveiling student academic resilience in language learning: a structural equation modelling approach

**DOI:** 10.1186/s40359-024-01665-1

**Published:** 2024-03-27

**Authors:** Siyu Duan, Xiaoyu Han, Xiaoxue Li, Honggang Liu

**Affiliations:** 1 School of Foreign Languages, Northeast Normal University, Changchun, Jilin Province 130024 China; 2 School of Foreign Languages, Soochow University, Soochow, Jiangsu Province 215006 China

**Keywords:** Academic resilience, English learning, Structural equation modelling approach, High school students

## Abstract

This paper aims to investigate the internal structure of student academic resilience in language learning, to assess its global and dimensional levels and to examine the gender and age-related differences. Therefore, 1,653 English as a foreign language (EFL) learners, including junior and senior high school students were selected as participants to complete the questionnaires. The results of exploratory and confirmatory factor analyses unveiled a four-factorial structure of student academic resilience in English learning, including positive individual characteristics, family support, teacher support and peer support. In addition, there were no significant differences between male and female students in terms of academic resilience in English learning and its four subdimensions. Only the level of family support for junior high school students was significantly higher than that of senior high school students. The findings, implications, and limitations of language learning academic resilience were discussed.

**Supplementary Information:**

The online version contains supplementary material available at 10.1186/s40359-024-01665-1.

## Introduction

Language learning is a process of socialisation [1, 2], wherein the performance of language learners depends not only on learners themselves but rather benefits from their interaction with their environment from an ecological perspective [3]. Language learners in school are likely to encounter a multitude of challenges and pressures, such as the high demands of teachers and parents and poor exam results, which could generate heightened levels of anxiety and demotivation and even disengagement and burnout when learning language [4, 5]. The research on student resilience provides valuable insight into how learners employ their positive individual characteristics (e.g. self-control, empathy) and contextual support (i.e. in-family and out-family support) to bounce back from challenges and setbacks. These factors serve as protective factors and integral components for fostering student resilience [6]. Academic resilience is usually viewed as a crucial ability to effectively manage the challenges encountered in academic learning and bounce back from adversity situations in the learning environment [7–10]. Research on student academic resilience research tends to treat it as a multidimensional construct [8, 11], highlighting its significant associations with critical protective factors, such as teacher efficacy beliefs and family communication patterns [9, 12]. However, little attention has been attached to exploring the internal structure of student academic resilience in language learning with both internal and external protective factors taken into consideration. The academic resilience scales used in China yet ignored the specific features among high school students [5, 13, 14]. Compared to other demographic groups, such as college students, high school students exhibit a more pronounced parental involvement and teacher support in their language learning process [15–17]. Moreover, they encounter unique challenges and unique pressures stemming from competition and exam-related stress like the national senior high school/college entrance examination. Lastly, the high school education phase represents a critical period for language acquisition that necessitates heightened attention towards student psychological states. Therefore, this study aimed to develop a domain-specific scale to measure academic resilience in English learning and validate its internal structure among high school students, and the resilience factors within the parental support and teacher support structure highlighted the crucial role of close connection of high school students with their family and teachers in nourishing student academic resilience.

## Literature review

### Understanding student academic resilience in language learning

In the “buffering processes” [6] (p.2) of withstanding language learning challenges and setbacks, resilience is usually viewed as, “[…] capacity for […] successful adaption despite challenging or threatening circumstances” [18] (p.426). It can motivate students to actively mobilize the protective factors to effectively navigate stress [4, 19] and create the conditions to bounce back from difficulties, thereby facilitating further development [6, 20]. In measuring student resilience, protective factors are of significance to interpret resilience [6], which includes individual internal characteristics (e.g., perseverance, empathy, sociability, persistence and self-regulation, self-control) [6, 11, 21] and external factors (family connection, teacher support) [6, 22]. During the language learning process, these internal and external protective factors facilitate students’ positive adaptation and recovery from academic setbacks or overcome risk factors that may hinder their language learning achievement [4, 14].

The manifestations of protective factors have been considered in measuring resilience in students’ language learning [4, 23]. Most of the prior studies focused on the internal features of learners, namely, the psycho-emotional characteristics in examining the multi-dimensional structure of resilience [8, 11, 21]. Cassidy’s [11] tri-factorial model delineated resilience into perseverance, reflecting and adaptive help-seeking and negative affect and emotional response. While, Kim and Kim [21] proposed a five-factorial structure, encompassing perceived happiness, empathy, sociability, persistence and self-regulation. Similarly, Martin and Marsh [8] advocated for a 5-C model of academic resilience: confidence (self-efficacy), coordination (planning), control, composure (low anxiety) and commitment (persistence). Recognizing the need to investigate student academic resilience in English learning, Liu and Han [4] and Wei et al. [23] adopted Kim and Kim’s [21] five-factorial framework to investigate the structure of academic resilience among Chinese senior high school and university students. Notably, self-regulation, as a core component, exhibited the highest contribution to fostering academic resilience in English learning [4, 23].

Despite its necessity and significance, the external protective factors (e.g., family, social factors such as teachers) are conspicuously missing from the instruments used to measure student academic resilience, despite evidence suggesting that student academic resilience emerges through students’ interaction with their social ecology, particularly within the family and the school [4, 12]. In summarizing and discussing the above factors, the current study specifically represents the attempt to delve into the inner structure of student academic resilience in language learning, with both internal and external protective factors taken into consideration.

Additionally, the consensus of variations in student academic resilience across socio-demographic variables, such as gender and age, has yet to be reached. Some research results have shown no significant differences in academic resilience across gender, age and year of study [7]. However, some studies have revealed significant differences in academic resilience, showing that girls are more resilient than boys [10]. By contrast, some studies have discovered that boys possess significantly stronger academic resilience than girls [24, 25]. Regarding age variables, the resilience of older students has evinced no significant difference from [7, 25] or proved to be lower than [10] that of younger students. Thus, these inconsistent findings motivated us to further explore the gender and age differences in students’ academic resilience in language learning.

### The hypothesised structure of student academic resilience in language learning

Typically, Lereya et al.’s [6] ten-factorial structure of resilience scale (including family connection, school connection, community connection, participation in home and school life, participation in community life, self-esteem, empathy, problem solving, goals and aspirations, and peer support) has been adopted frequently in student academic resilience research in the language education field [5, 13, 14]. In these studies, Lereya et al.’s [6] framework served as a research instrument to measure the role of academic resilience in learners’ engagement [5] and anxiety [13] or the relationship between academic resilience and academic motivation [14]. The attention given to this scale might be attributed to the holistic perspective on resilience presented by Lereya et al. [6], which encompasses positive individual characteristics and external supports.

The language learning process is a dynamic interaction with external support, providing scaffolding for learners to confront academic adversities and setbacks [15, 26] from family [16], school [27] and peers [28]. Accordingly, Lereya et al.’s [6] ten-factorial structure was adopted as the framework for this study, but the community factors, such as community connection and participation in community life, were excluded as the school and family are the primary domains for language learning in China. The items within the family connection factor in this framework can be regarded as pertaining to parental emotional support based on their semantic connotations. Indeed, parental behavioural support is a crucial factor for student academic performance and exerts a positive predictive effect on student psychology [16, 17]. Thus, both parental behavioural support and parental emotional support were taken into consideration in family support [16, 17]. Simultaneously, recognising the pivotal role of teachers in the school as significant contributors to student psychology, academic performance and holistic development [15, 26, 29, 30], teacher support was also examined in the current study.

Accordingly, the preceding discussion yielded a hypothetical structure of student academic resilience in English learning, including self-esteem, empathy, goals and aspirations and problem-solving (four positive individual characteristics); parental emotional support and behavioural support (both pertaining to family support); teacher support; and peer support.

Therefore, the study addressed the following research questions (RQs):

#### RQ1

What is the structure of student academic resilience in English learning?

#### RQ2

What are the global and dimensional levels of student academic resilience in English learning?

#### RQ3

Are there any gender- and age-related differences in student academic resilience in English learning?

## Methods

### Research participants

Altogether 1,806 Chinese high school students were invited to participate in this study. The participants were mainly from the northern and eastern areas of China, such as Jilin, Shandong and Jiangsu provinces. All participants were learning English as a foreign language. A valid sample of 1,653 students was obtained after screening for invalid responses. Among them, 666 (40.3%) were male and 987 (59.7%) were female; 789 (47.7%) were junior high school students (*M*_*age*_ = 13.1 years) and 864 (52.3%) were senior high school students (*M*_*age*_ = 16.3 years). The grade levels of the students were closely associated with their age since their age generally increased when entering schools of a higher age. In this study, it means that younger students are assigned to lower grades (i.e. junior high school) while older students are placed in higher grades (i.e. senior high school).

### Research instrument

The questionnaire comprised two sections. The first part collected the participants’ demographic information, including gender, age, grade level and area. The second section was the Student Academic Resilience in English Learning Scale (SARELS) (see Appendix E for items), developed based on the hypothesised structure proposed in Sect. 2.2. The items were mainly from Lereya et al.’s [6] Resilience Scale, the other items came from other scales (i.e. Elliot and Murayama [31], Liu’s [16]^1^, Liu and Li’s [15], Kim and Kim’s [21], and Liang’s [32]). The scale applied an eight-factorial design, encompassing self-esteem (*n =* 3), empathy (*n =* 4), problem-solving (*n =* 4), goals and aspirations (*n =* 4), parental emotional support (*n =* 3), parental behavioural support (*n =* 5), teacher support (*n =* 6) and peer support (*n =* 4). Specific information about the designed questionnaire is available in Appendix A.

### Data collection

The convenience sampling method was employed in this study to collect the data giving easy access to the research participants [33]. In May 2023, we invited some acquainted English teachers to share the questionnaire with their students via the online survey platform of Wenjuanxing (https://www.wjx.cn/). The respondents were made clear of the research purposes and assured of voluntary participation and confidentiality of their responses before answering the questionnaires. They were motivated to give honest answers to the questions.

The Wenjuanxing system’s timing records indicated that the average answer time for each question was five seconds. Therefore, responses that were provided faster than this were disregarded. Along with this, the Mahalanobis distance was calculated to weed out outliers. After eliminating 153 outliers, we obtained 1,653 legitimate responses for the data analysis.

### Data analysis

IBM SPSS Statistics 26.0 and IBM Amos 24.0 were used to record and analyse all the quantitative data. For RQ1, the inner structure of students’ academic resilience in English learning in the Chinese context was examined by conducting EFA and CFA. The sample was randomly split into two parts using the SPSS software, with Dataset 1 being used for the EFA (*N =* 842) and Dataset 2 for the CFA (*N =* 811). First, we tested the sample’s univariate normality and measured each item’s discrimination. Next, principal axis factoring was applied to probe the inner structure of students’ academic resilience in English learning, with confirmation results received from IBM Amos 24.0. In addition, multiple-group analysis was conducted to examine the measurement invariance of the construct across gender and age levels in IBM Amos 24.0. After validating the stability of the two baseline models, indicators including configural invariance (M1), measurement weights invariance (M2) and structural covariance invariance (M3) were compared. To this end, the values of ΔNFI, ΔIFI, ΔRFI and ΔTLI in the *χ*^*2*^ difference tests between M2 and M1, and M3 and M2 were taken into consideration [34]. For RQ2, descriptive analysis was performed to investigate students’ academic resilience levels in English learning. For RQ3, an independent samples t-test was conducted to check for any significant gender or age-related differences in students’ academic resilience in English learning.

## Results

### The structure of student academic resilience in English learning

#### Normality and item analysis results

We first carried out tests of univariate normality to ensure the normal distribution of the data. The results indicated that the collected data (*N =* 1,653) were normally distributed as the values of skewness and kurtosis met the benchmarks of less than |3.0| and |10.0|, respectively [35]. Next, the discriminant validity of all items was guaranteed via item analysis [36]. In detail, the independent samples t-test was performed to compare the 27% highest- and 27% lowest-scoring surveyed participants [37]. Every item showed a significant difference between the two groups (*p* < 0.01), and therefore all items were kept for later analysis. Subsequently, we employed item-total correlation analysis to delve into the correlation between every item and the global scale. All items met the correlation coefficient benchmark (*r* > 0.30, *p* < 0.01) [36].

#### Results of the exploratory factor analysis

The Kaiser-Meyer-Olkin measure of sampling adequacy was 0.947 (close to 1), and the results of Bartlett’s test of sphericity were *χ*^*2*^ = 17,904.869, *df* = 528, *p* = 0.000,^2^ indicating the sufficient correlation between variables and the acceptability of the data for the follow-up factor analysis [36]. The 33 items were then handled by principal axis factoring analysis, the outcomes of which are displayed in Table 1. Items whose factor loadings were less thanc|0.4|, cross-loadings greater than |0.4|, cross-loading difference less than |0.2| or a commonality value below 0.4 were eliminated [38]. In the end, six factors were identified, and five items (PC09, PC10, PC14, PC15 and FS05) were deleted in compliance with Hair et al.’s [38] suggestions. The cumulative percentage of total variance explained by the six identified factors was 59.232%, which was higher than the EFA referential line of 55% (cf. Plonsky and Gonulal [39]), demonstrating that the obtained factors were satisfactory.

**Table 1:** Results of EFA (Pattern matrix^ɑ^)

	Factor1 Positive Individual Characteristics	Factor2 Teacher Support	Factor3 Family Support1	Factor4 Peer Support	Factor5 Problem-solving	Factor6 Family Support2	Commu nalities
PC04 I can lead conversations well in accordance with a specific atmosphere or interlocutor.	**0.867**	0.059	0.085	-0.011	-0.105	0.038	0.814
PC03 I am good at finding the right words for what I would like to express.	**0.835**	0.072	0.088	-0.017	-0.085	-0.032	0.767
PC02 There are many English tests on which I do well.	**0.788**	0.038	0.000	-0.025	0.089	-0.019	0.738
PC08 I can recognise how people feel by their facial expressions.	**0.704**	0.038	0.063	-0.035	0.040	-0.005	0.621
PC01 I can do most English exercises if I try.	**0.650**	0.062	-0.021	-0.142	0.117	-0.030	0.659
PC07 I can work out my English problems.	**0.649**	0.040	0.045	-0.203	0.099	-0.102	0.730
PC06 I have goals and plans for future English learning.	**0.546**	-0.005	0.099	-0.127	0.219	0.098	0.685
PC13 My aim is to perform well relative to other students in English.	**0.376**	-0.056	0.015	0.031	0.221	0.069	0.256
TS03 The English teacher pays careful attention to my studies.	-0.014	**0.785**	-0.030	-0.013	0.038	0.007	0.623
TS05 The English teacher helps me choose suitable learning materials.	0.095	**0.781**	-0.067	-0.044	-0.139	0.106	0.544
TS06 The English teacher helps me choose suitable extra-curricular reading materials.	0.103	**0.758**	-0.024	-0.030	-0.110	0.061	0.621
TS01 The English teacher shows us how to compensate for limited knowledge (such as attributive clauses, etc.).	-0.028	**0.645**	0.057	0.002	0.062	-0.122	0.446
TS04 The English teacher has high expectations of me.	0.130	**0.642**	0.009	0.029	0.069	0.054	0.528
TS02 The English teacher imparts practical knowledge to us (such assentence patterns, etc.).	-0.130	**0.640**	0.074	-0.043	0.098	-0.080	0.456
FS08 My parents set mean example with their own experiences in learning English.	0.072	-0.035	**0.877**	-0.019	-0.062	-0.035	0.777
FS04 My parents assist me in learning English.	0.041	-0.028	**0.787**	-0.005	-0.065	0.012	0.621
FS03 My parents encourage me when I make progress in learning English.	-0.049	0.153	**0.414**	-0.088	0.212	0.096	0.427
FS01 At home, there is an adult who is interested in my English school work.	0.119	0.102	**0.332**	-0.084	0.165	0.252	0.503
PS04 My classmates would point out my mistakes in English learning and encourage me.	0.025	0.018	-0.015	**-0.934**	-0.112	-0.003	0.836
PS03 My classmates would share English learning resources with me.	0.052	0.023	0.018	**-0.843**	-0.048	0.022	0.771
PS02 My classmates would pick me for a partner when dealing with English tasks (e.g., role playing, classroom activities).	0.101	-0.030	0.036	**-0.786**	-0.011	-0.014	0.699
PS01 My classmates would make me feel better when I have difficulties in learning English.	-0.096	0.008	0.008	**-0.761**	0.116	-0.002	0.592
PC05 When I encounter words and sentence patterns I don’t know in English, I look them up in a dictionary and look for references.	0.209	0.061	0.034	-0.029	**0.681**	-0.019	0.717
PC12 When I encounter problems in my English studies, I will go online to search for relevant resources.	0.174	0.101	-0.048	-0.114	**0.519**	0.059	0.522
PC11 I try to work out English problems by talking about them with my teacher and classmates.	0.270	0.135	0.020	-0.287	**0.342**	0.045	0.659
FS07 My parents afford to tutor me in English.	-0.081	-0.029	-0.044	-0.024	0.002	**0.508**	0.242
FS06 My parents made an effort to find a way to enroll me in the class I am currently attending.	0.098	-0.007	0.047	0.037	-0.029	**0.483**	0.263
FS02 At home, there is an adult who wants me to do my best to learn English.	-0.057	0.157	0.220	-0.058	0.094	**0.388**	0.367
Total Variance Explained Cumulative%	38.391	45.739	50.531	54.742	57.481	59.232	——
Cronbach *α*	0.927	0.860	0.752	0.907	0.852	0.841	——

Note. Extraction method: Principal axis factoring

Rotation Method: Oblimin with Kaiser normalisation ^a^

^a^Rotation converged in nine iterations

The six factors were named positive individual characteristics, teacher support, family emotional support, peer support, problem-solving and family behavioural support. Factor 1, positive individual characteristics, covers empathy (PC03, PC04, PC08), self-esteem (PC01, PC02, PC07) and goals and aspirations (PC06, PC13). Factor 2, teacher support, describes teachers’ provision of academic instruction (TS01, TS02), tangible assistance (TS05, TS06) and emotional care (TS03, TS04). Factor 3, family support 1, includes items FS01, FS03, FS04 and FS08. Factor 4, peer support, entails PS01, PS02, PS03 and PS04. Factor 5, problem-solving, involves PC05, PC11 and PC12. Factor 6, family support 2, includes FS02, FS06, and FS07.

#### Results of the confirmatory factor analysis

**Fig. 1:**
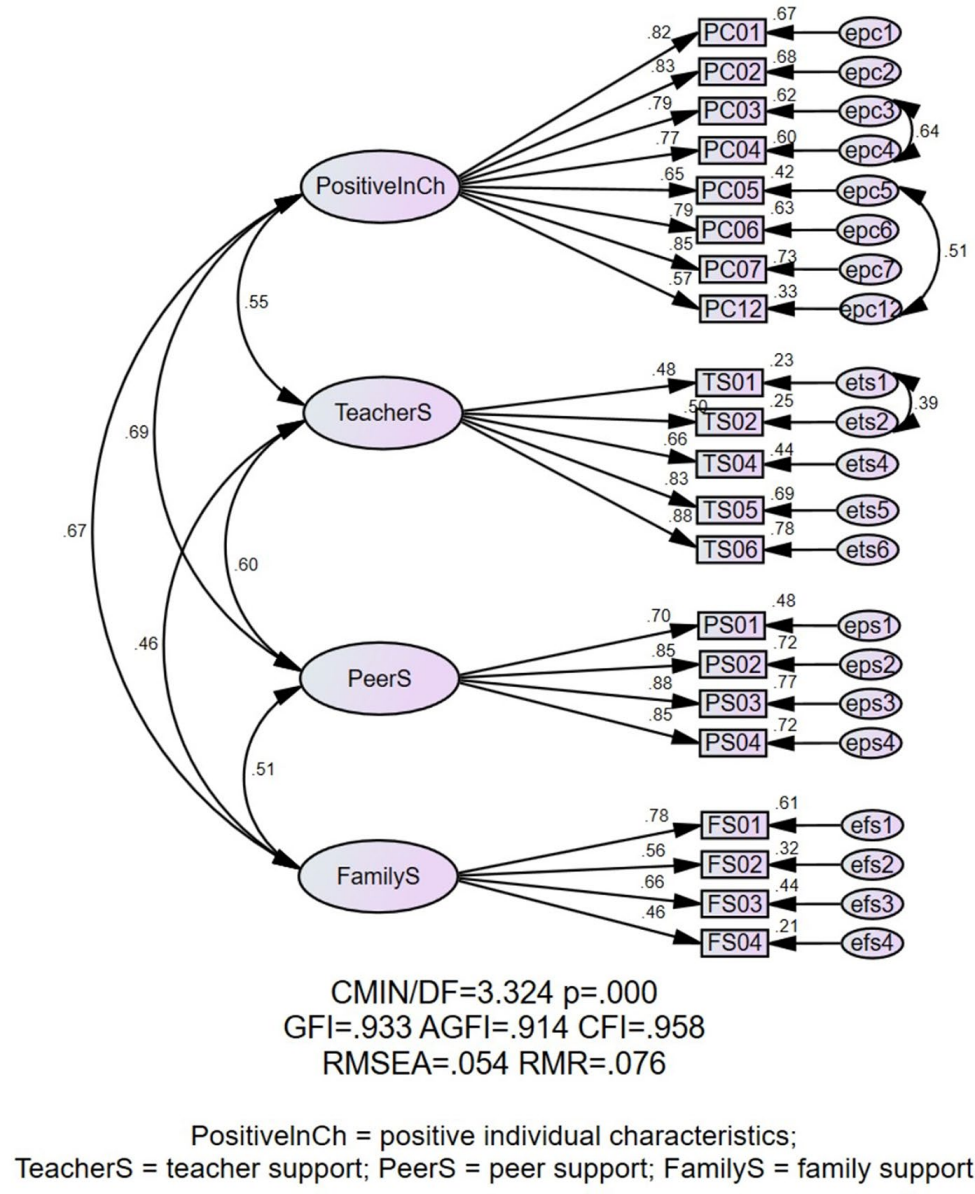
The final model of students’ academic resilience in English learning

We ran a CFA (*N =* 811) to examine whether the obtained structure of student academic resilience in English learning fit the data in the current sample. CFA was performed on the subdimensions and then the whole measurement model was explored. Among those subdimensions, the discriminant validity of factors 1 and 5 was unacceptable. To be more specific, the correlation coefficients between factors 1 and 5 (0.80) were larger than the square root of the AVE values of them (0.77 and 0.79) [34]. As both dimensions concerned positive characteristics, the related items were merged into one dimension. The same applied to factors 3 and 6, which were both related to family support and exhibited unacceptable discriminant validity (the correlation coefficients between factors 3 and 6 [0.75] were larger than the square root of the AVE values for them [0.70 and 0.52]) [34]. According to the benchmarks proposed by Kline [35] for indices assessing model fit (*χ*^*2*^/*df* ≤ 8; GFI ≥ 0.90; AGFI ≥ 0.90; CFI ≥ 0.90; RMSEA ≤ 0.08; SRMR ≤ 0.1), the CFA results confirmed that the whole measurement model showed a good fit (see Fig. 1). Specifically, *χ*^*2*^/*df*, RMSEA and SRMR were 5.798, 0.077 and 0.043, respectively, and GFI, AGFI and CFI were all greater than 0.90. The values reached the cut-off scores mentioned above.

Four factors were obtained from the CFA. Based on the results of the EFA and CFA, four factors emerged and labelled as positive individual characteristics (PC01, PC02, PC03, PC04, PC05, PC06, PC07, PC12), teacher support (TS01, TS02, TS04, TS05, TS06), peer support (PS01, PS02, PS03, 0S04) and family support (FS01, FS02, FS03, FS04).

We also calculated convergent and discriminant validity to confirm the validity of the SARELS. The results of these calculations can be found in Appendix B. Regarding convergent validity, the average variance extracted (AVE) values were higher than 0.5 and the composite reliability (CR) values were higher than 0.7, demonstrating the good convergent validity of the model [38]. When assessing discriminant validity, one consideration is the comparison of the square root value of the AVE and the correlation coefficients of each scale [38]. As shown in Appendix B, the square root value of the AVE of subscales was higher than their corresponding correlation coefficients (*r*), suggesting the good discriminant validity of each subscale.

We used IBM Amos 24.0 to determine the measurement invariance of the four-factorial structure of students’ academic resilience in English learning across genders. To this end, we first established two baseline models to identify if the four-factorial model fit the data well for both the male and female groups by running individual CFAs, a prerequisite of multi-group analysis. Second, the models were tested against less-constrained models to examine the stability of the model with the following indices: configural invariance (M1), measurement weights invariance (M2), and structural covariance invariance (M3).

The results are shown in Table 2. The model fit indices for the two gender groups were similar, indicating that a deeper multi-group analysis was possible. Furthermore, the results of the multi-group analysis show that the *χ*^*2*^ difference tests between M2 and M1, M3 and M2 were not significant since the ΔNFI, ΔIFI, ΔRFI and ΔTLI were below 0.05 [34]. The results indicated that the obtained structural equation model was invariant for both gender groups.

**Table 2:** Results of multi-group analysis of the SEM model across gender

	χ^2^	df	χ^2^/df	GFI	AGFI	CFI	RMSEA	SRMR	Δχ^2^ (Δdf)
Male	382.451	180	2.215	0.895	0.865	0.945	0.060	0.057	——
Female	469.517	180	2.608	0.915	0.891	0.956	0.057	0.044	——
M1	852.030	360	3.367	0.907	0.881	0.952	0.041	0.057	——
M2	865.061	377	2.295	0.906	0.885	0.952	0.040	0.059	13.031(17)
M3	888.161	387	2.295	0.903	0.885	0.951	0.040	0.064	23.100* (10)

Note *p < 0.05; *N =* 811

Parallel processes were also undertaken to examine the measurement invariance of the four-factorial structure of students’ academic resilience in English learning across ages. For convenience, the junior high school students (*M*_*age*_ = 13.1 years) were treated as the younger group, and the senior high school students (*M*_*age*_ = 16.3 years) as the elder group. From Table 3, we can see that the model fit indices for the two baseline models of each age group were similar, indicating the feasibility of further multi-group analyses. Furthermore, the results of the multi-group analysis show that the *χ*^*2*^ difference tests between M2 and M1, M3 and M2 were not significant since the ΔNFI, ΔIFI, ΔRFI and ΔTLI were below 0.05 [34], suggesting that the obtained structural equation model was invariant for both age groups.

**Table 3:** Results of multi-group analysis of the SEM model across age

	χ^2^	df	χ^2^/df	GFI	AGFI	CFI	RMSEA	SRMR	Δχ^2^ (Δdf)
Younger Group	443.914	180	2.466	0.901	0.873	0.944	0.061	0.052	——
Elder Group	454.218	180	2.523	0.905	0.879	0.951	0.060	0.045	——
M1	898.132	360	2.495	0.903	0.876	0.948	0.043	0.052	——
M2	923.936	377	2.451	0.901	0.879	0.947	0.042	0.053	25.804 (17)
M3	931.675	387	2.407	0.900	0.881	0.947	0.042	0.053	7.738(10)

*Note N =* 811

### Levels of student academic resilience in English learning

We assessed the levels of students’ academic resilience in English learning in the Chinese context at both the general and dimensional levels, the results of which were shown in Table 4. More specifically, students reported a moderate to high level of academic resilience in English learning (*M* = 4.71, *SD* = 0.86). To be more specific, students experienced a high level of teacher support (*M* = 5.33, *SD* = 0.79), followed by peer support (*M* = 4.71, *SD* = 1.26), positive individual characteristics (*M* = 4.53, *SD* = 1.11) and family support (*M* = 4.30, *SD* = 1.12) at a moderate to high level.

**Table 4:** The levels of students’ academic resilience in English learning

Dimension	Min	Max	M	SD	Skewness	Kurtosis
Positive Individual Characteristics	1.00	6.00	4.53	1.11	−0.40	−0.52
Teacher Support	1.40	6.00	5.33	0.79	−1.24	1.17
Peer Support	1.00	6.00	4.71	1.26	−0.81	−0.08
Family Connection	1.00	6.00	4.30	1.12	−0.34	−0.29
Global Resilience	2.14	6.00	4.71	0.86	−0.34	−0.65

*NoteN* = 811

### Differences in student academic resilience in English learning in terms of gender and age

We conducted an independent samples t-test using IBM SPSS Statistics 26.0 and calculated the effect size to examine whether there were any significant differences in students’ academic resilience in English learning in terms of gender (see Appendix C) and age (see Appendix D). The effect size index, Hedges’ *g*, from the *d*-family, was used. According to Cohen [40], a Hedges’ *g* value equal to or lower than 0.20 indicates a weak effect size, a Hedges’ *g* value higher than 0.20 and equal to or lower than 0.50 indicates a moderate effect size and a Hedges’ *g* value higher than 0.50 indicates a strong effect size. To mitigate the effects of gender imbalance in the sample (male students = 314, female students = 497), we randomly selected about 60% of the female students using SPSS software and constructed a new sample (male students = 314, female students = 308). The results indicated no significant differences between male and female students in terms of positive individual characteristics, teacher support, peer support, family support and global resilience.

The results indicated that junior high school students reported significantly higher family support than senior high school students with a trivial effect size (*t* (809) = 2.341, *p* < 0.05, Hedge’s *g* = 0.170). No significant differences were found between junior and senior high school students regarding positive individual characteristics, teacher support, peer support and global resilience.

## Discussion

### Structure of student academic resilience in English learning

Based on Lereya et al.’s [6] resilience framework, this study validated our hypothesised four-factorial structure, including positive individual characteristics, family support, teacher support and peer support (presented in Sect. 2.2). The findings provide further evidence for the multidimensional structure of student academic resilience in language learning, despite minor variations in the terminology used to describe its components [4, 21, 23].

Factor 1 is positive individual characteristics, including the components in the resilience model of Lereya et al. [6] – namely, empathy (PC03, PC04), self-esteem (PC01, PC02, PC07), problem-solving (PC05, PC12) and goals and aspirations (PC06). There is additional evidence substantiating that these elements, such as empathy, are integral components of academic resilience [4, 21]. Empathy is the ability to comprehend and share feelings and emotions with others, to empower students to better adapt in challenging language learning situations [21]. Self-esteem pertains to the individual’s self-assessment of their competence, believing in their ability to effectively handle tasks [41] and exhibiting confidence when confronted with challenges [42]. Problem-solving and goals and aspirations have been identified as resilience factors and negatively associated with student depression [43]. Together, these factors contribute to promoting students’ positive adaptation to effectively cope with academic challenges and stresses and are therefore considered components of academic resilience. Furthermore, the “EMPATHICS” version of well-being, from the perspective of positive psychology, aligns with our expectations to view these factors as positive individual characteristics. Empathy and self-esteem are essential components of the “EMPATHICS” version of well-being, while goals and aspirations hold intrinsic significance for individuals [44]. Although the “EMPATHICS” version of well-being does not explicitly propose problem-solving, its associations with self-esteem and agency prompted us to consider problem-solving as a positive individual characteristic. Accordingly, factor 1 is named positive individual characteristics.

Factor 2 is teacher support, describing how a teacher “helps, befriends, trusts, and is interested in students” [45] (p. 503). In this study, it entails academic instruction (TS01, TS02), tangible assistance (TS05, TS06) and emotional care (TS04). Within the school environment, where students spend most of their time, teachers provide different types of support, such as academic support, emotional support and instrumental support [15, 26, 30]. The existing research outlines that language teacher support may matter in facilitating student engagement in language learning, fostering their confidence and enhancing their ability to effectively manage challenges in highly demanding learning situations [15, 30]. In general, the findings of this study suggest that students who perceived more teacher support exhibit stronger academic resilience.

Factor 3 is peer support (PS01, PS02, PS03, PS04), which has been identified as a crucial component of resilience according to Lereya et al. [6]. It refers to students’ feeling of receiving emotional care, companionship and help through interacting with their peers [46]. The reason for the much attention paid to the peer support component of resilience might emanate from its catalytic role in facilitating students’ effective coping with academic challenges. For one thing, students perceived peer support can mitigate the risk of academic failure and improve their subjective satisfaction through the provision of emotional and informational support [47], as well as affording and stimulating additional learning opportunities [28]. For another, peer academic support (e.g. providing information and comparing learning situations with each other) can enhance students’ learning motivation [46], which has been acknowledged as a crucial factor contributing to academic resilience in achieving future language learning success [14].

Factor 4 is family support, which mainly involves communication, understanding and parental investment [12, 16], entailing emotional support (FS01, FS03, FS04) and behavioural support (FS02) in this study. Notably, family support is frequently attained through family investments, which is a multidimensional construct encompassing parental investment beliefs (i.e. active investment belief and non-investment belief) and parental investment behaviours (i.e. knowledge investment behaviour, relationship investment behaviour, emotional investment behaviour and economic investment behaviour). These dimensions were empirically demonstrated to exert an influence on students’ positive psychology in Liu’s [17] study. This result also agrees with the survey result that family support contributes to student academic resilience [12]. On one hand, family emotional support fosters students’ confidence, facilitates positive adaptation and enables the effective resolution of challenges encountered in one’s personal life and academic studies [48]. On the other hand, family behavioural support significantly contributed to student academic motivation, attitude and performance [16], which are the key protective factors in promoting academic resilience.

### Levels of student academic resilience in English learning

The data analysis results evinced students’ academic resilience in English learning was moderate to high, which aligns somewhat with the findings of Liu and Han’s previous study [4]. This finding suggested that the majority of students exhibited a moderate to high level of academic resilience when confronted with academic adversities and challenges in English learning, owing to their positive individual characteristics, family support, peer support and teacher support. Among the subdimensions, EFL learners in China exhibited a high level of teacher support and a moderate to high level of peer support, positive individual characteristics and family support.

One possible reason for the highest level of teacher support can be related to the psychological state of the students. Students with sound psychological conditions, for example, with grittier personalities, higher well-being and lower anxiety tend to experience high levels of teacher support [26, 49]. In addition, the English learning environment may also exert an influence. The frequent teacher-student interaction and communication during the lesson contribute to establishing a harmonious learning environment [15, 30]. Under such a circumstance, teacher support can be considered a form of performance promotion [50], thereby promoting students’ positive attitude and efficiency in language learning [51].

The moderate to high level of peer support of academic resilience in English learning also reflects the interactive nature of language learning. Language learners spend a substantial amount of time interacting with their peers, who may encounter similar academic challenges. Thus, peer interaction has the potential to cultivate students’ confidence in their academic abilities and promote their active engagement in learning [52]. In addition, peer support has been shown to facilitate learners’ positive adjustment [46] and language development [28].

The assessment of positive individual characteristics enables us to gain insights into their beneficial impact on students’ ability to rebound from academic adversities [6, 21]. Positive individual characteristics students positive behavioural adaptation and academic success, and it is of great significance for students to overcome adversity and concentrate on their future goals [4, 6, 21]. Resilient students, therefore, exhibit a stronger sense of positive individual characteristics compared to their less resilient counterparts [21], as they attribute their language achievements to their personal abilities and internal qualities [4].

Family support, primarily through parental behavioural and emotional support, is critical in facilitating students’ emotional regulation, academic resilience and academic achievement. Adolescence is a vital period of individual development that is characterised by a multitude of physiological, psychological and social changes. High school students encounter various complex development challenges during this pivotal stage in their studies and lives, with family support assuming paramount significance. Numerous studies [12, 16] have demonstrated that family support significantly enhanced student academic engagement and performance.

### Differences in student academic resilience in English learning in terms of gender and age

The data analysis showed no statistically significant gender differences in academic resilience and its subdimensions among high school students in English learning. This result is in accordance with academic research findings in general education [7]. This may be attributed to the current coeducation in Chinese high schools, which adopt a unified teaching approach in daily instruction. Following the principles of humanistic education, curriculum standards prioritise students’ knowledge acquisition during teaching without emphasising gender differences or gender prejudice to create a harmonious and independent learning atmosphere for boys and girls.

Regarding differences in age level, there were no significant differences between junior and senior high school students in global academic resilience, positive individual characteristics, teacher support and peer support. Only the level of family support among junior high school students was significantly higher than that observed among senior high school students. This result is particularly relevant for two reasons. First, as students grow older, the role of family behavioural support in their academic development diminishes, potentially due to escalating task demands. As students advance to the senior level, the curriculum becomes increasingly challenging for parents who may lack the capacity or sufficient knowledge to comprehend it. The second reason for the relevance of this result may lie in the enhancement of self-regulation among senior high school students, which has been recognized as a component of student academic resilience [4, 21]. This implies that students demonstrate proficiency in regulating their emotions, resulting in decreased dependence on family emotional support.

## Conclusion and Implications

An attempt was made in this study to examine the structure and level of student academic resilience and differences in the socio-demographic variables of gender and age. The study designed and validated a scale to measure student academic resilience in English learning within the Chinese context, mainly adopting the framework of Lereya et al. [6]. We confirmed that student academic resilience in English learning is a four-factorial structure comprising positive individual characteristics, family support, teacher support and peer support. In addition, the level of global academic resilience was moderate to high, with the teacher support level at the highest level, followed by peer support, positive individual characteristics and family support. Regarding differences in the socio-demographic variables of gender and age, the only dimension that showed a difference based on age level was family support.

The results of this study underscore the significance of individual traits, family support, teacher support and peer support as integral components of student academic resilience. These findings serve as a catalyst for future language teaching and learning with the following implications. First, it is established that teachers and parents should give way to cultivate and sustain positive psychological qualities in students alongside their academic achievements. Research has demonstrated that fostering these positive psychological qualities, such as self-esteem, self-efficacy, and empathy, not only helps students cope with stress and challenges in life but also contributes to maintaining optimal learning motivation and enthusiasm, enhancing their academic performance [4, 7, 8, 21, 53] even the life-long learning. Furthermore, the support from significant others should be juxtaposed with positive individual characteristics in fostering student academic resilience. Teachers, families and peers – as influential factors closely connected with students – play a crucial role in cultivating academic resilience through their emotional and behavioural support. Therefore, it is imperative for teachers, families and peers to reinforce contact with students and empower them to establish harmonious relationships and create an optimal learning atmosphere.

The scale developed and validated in this study exhibited high reliability and validity; however, the study had four limitations. First, possibly expanding the participant pool beyond urban areas to encompass students from rural areas would provide a more representative sample, facilitating broader generalization of findings. Second, the gender imbalance needs to be undertaken in future studies to reduce the uneven sampling due to gender differences. Third, we primarily utilized quantitative research methods by designing a comprehensive questionnaire. In future studies, it would be advantageous to incorporate a more extensive range of qualitative data and ensure the study’s reliability and validity through triangulation. Lastly, with the ecological turn in language education, the study of language teaching and learning from an ecological perspective has been burgeoning [54–57]. Notably, the four factors identified in this study as contributing to student academic resilience pertain to the personal system and microsystem [58]. However, given the dynamic nature of an ever-changing ecosystem, it is foreseeable that student academic resilience should also encompass additional significant components within the broader ecological context, such as curriculum-related factors in the exosystem and cultural factors in the macrosystem.

### Electronic supplementary material

Below is the link to the electronic supplementary material.

Supplementary Material 1

## Data Availability

The datasets and materials used and/or analysed during the current study are available from the corresponding author on reasonable request.

## Footnotes

1 Please refer to Liu [17] for the bilingual version (English and Chinese) of the questionnaire.

2 According to Field [36], the Kaiser-Meyer-Olkin and Bartlett’s test of sphericity are significant indices for measuring correlations between variables. Specifically, a value close to 1 for Kaiser-Meyer-Olkin (which varies between 0 and 1) and a significance value below 0.05 for Bartlett’s test suggest a strong correlation between variables.
