# Supplementary material for: Unveiling student academic resilience in language learning: a structural equation modelling approach
Source: BMC Psychol. 2024 Mar 27;12:177. doi: 10.1186/s40359-024-01665-1 (PMC10976839; doi:10.1186/s40359-024-01665-1)
Supplement: Supplementary file 1 — Supplementary Material 1 [file 40359_2024_1665_MOESM1_ESM.docx]

# Appendix A. Detailed information on student academic resilience in English learning scale.

| **Dimensions** | **Items Distribution** | **Sources** |
| --- | --- | --- |
| peer support | PS01, PS02, PS03, PS04 | Lereya et al. (2016); Liang (2017) |
| teacher support | TS01, TS02, TS03, TS04, TS05, TS06 | Liu & Li (2023) |
| self-esteem | PC01, PC02, PC07 | Lereya et al. (2016) |
| empathy | PC03, PC04, PC08, PC09 | Lereya et al. (2016); Kim & Kim (2017) |
| problem solving | PC05, PC10, PC11, PC12 | Lereya et al. (2016) |
| goals and aspirations | PC06, PC13, PC14, PC15 | Elliot & Murayama (2008); Lereya et al. (2016) |
| family connection (emotional support) | FS01, FS02, FS03 | Lereya et al. (2016) |
| behavioral support | FS04, FS05, FS06, FS07, FS08 | Liu (2013, 2024)^[[1]](#footnote-0)^ |

# Appendix B. Convergent and discriminant validity of each dimension.

| Factor | Item | **Convergent Validity** | | | **Discriminant Validity (*r*)** | | | | | |
| --- | --- | --- | --- | --- | --- | --- | --- | --- | --- | --- |
|  |  | *p* | CR | AVE | Positive Individual Characteristics | Teacher Support | Peer Support | | Family Support | |
| **Positive Individual Characteristics** | PC01, PC02, PC03, PC04,  PC05, PC06, PC07, PC12 | 0.000 | 0.917 | 0.585 | **0.765** |  | |  | |  |
| **Teacher Support** | TS01, TS02, TS04, TS05, TS06 | 0.000 | 0.810 | 0.475 | 0.515** | **0.690** | |  | |  |
| **Peer Support** | PS01, PS02, PS03, PS04 | 0.000 | 0.892 | 0.675 | 0.628** | 0.533** | | **0.822** | |  |
| **Family Support** | FS01, FS02, FS03, FS04 | 0.000 | 0.715 | 0.395 | 0.554** | 0.355** | | 0.413** | | **0.628** |

*Note.* The bold number in discriminant validity represents the square root value of AVE.

# Appendix C. Differences in resilience in English learning in terms of students’ gender.

|  | Male  (*n* = 314) | | Female  (*n* = 308) | | MD | *t* (620) | Hedges’ *g* |
| --- | --- | --- | --- | --- | --- | --- | --- |
|  | M | SD | M | SD |  |  |  |
| Positive Individual Characteristics | 4.56 | 1.19 | 4.51 | 1.09 | 0.045 | 0.488 | 0.044 |
| Teacher Support | 5.40 | 0.78 | 5.31 | 0.78 | 0.084 | 1.344 | 0.115 |
| Peer Support | 4.67 | 1.33 | 4.74 | 1.25 | -0.069 | -0.666 | 0.054 |
| Family Support | 4.30 | 1.19 | 4.31 | 1.04 | -0.012 | -0.129 | 0.009 |
| Global Resilience | 4.73 | 0.91 | 4.71 | 0.84 | 0.022 | 0.307 | 0.023 |

# Appendix D. Differences in resilience in English learning in terms of students’ age.

|  | Junior High School Students (*n* =393) | | Senior High School Students (*n* =418) | | MD | *t* (809) | Hedges’ *g* |
| --- | --- | --- | --- | --- | --- | --- | --- |
|  | M | SD | M | SD |  |  |  |
| Positive Individual Characteristics | 4.53 | 1.07 | 4.53 | 1.15 | -0.001 | -0.008 | 0.000 |
| Teacher Support | 5.30 | 0.79 | 5.36 | 0.80 | -0.061 | -1.091 | 0.075 |
| Peer Support | 4.70 | 1.25 | 4.71 | 1.28 | -0.013 | -0.142 | 0.008 |
| Family Support | 4.40 | 1.11 | 4.21 | 1.12 | 0.183 | 2.341^*^ | 0.170 |
| Global Resilience | 4.72 | 0.84 | 4.70 | 0.89 | 0.018 | 0.293 | 0.023 |

*Note.* **p* < 0.05.

# Appendix E. Student Academic Resilience in English Learning Scale (SARELS).

| Items (in English) | Items (in Chinese) |
| --- | --- |
| PC01 I can do most English exercises if I try. | 只要我努力，大部分英语习题我都能做到。 |
| PC02 There are many English tests on which I do well. | 有很多英语测验我都能做得很好。 |
| PC03 I am good at finding the right words for what I would like to express. | 我善于找到合适的英语词汇来表达自己的想法。 |
| PC04 I can lead conversations well in accordance with a specific atmosphere or interlocutor. | 英语交流中，我能够根据特定的情境和对话者，很好地引导对话。 |
| PC05 When I encounter words and sentence patterns I don’t know in English, I look them up in a dictionary and look for references. | 当我英语学习遇到不会的单词、句型的时候，我会查字典、找参考资料。 |
| PC06 I have goals and plans for future English learning. | 我有长期的英语学习目标与计划。 |
| PC07 I can work out my English problems. | 我可以解决英语学习过程遇到的问题。 |
| PC08 I can recognise how people feel by their facial expressions. | 我能从别人的面部表情感知他们的感受。 |
| PC09 When my friends are angry, I readily find out the reason for that. | 当我的朋友们生气时，我能很容易找到原因。 |
| PC10 I know where to go for help when I have an English problem. | 当我英语学习遇到困难时，我知道去哪里寻求帮助。 |
| PC11 I try to work out English problems by talking about them with my teacher and classmates. | 当我英语学习遇到困难时，我会尝试和老师、同学沟通来解决问题。 |
| PC12 When I encounter problems in my English studies, I will go online to search for relevant resources. | 当我英语学习遇到不会的问题时，我会到网上找相关资源。 |
| PC13 My aim is to perform well relative to other students in English. | 我的目标是英语成绩比大多数人好。 |
| PC14 I am striving to understand the content of English course as thoroughly as possible. | 我努力去尽可能透彻得理解英语课堂的内容。 |
| PC15 My goal is to learn English as much as possible. | 我的目的是在英语课程中学到尽可能多的东西。 |
| TS01 The English teacher shows us how to compensate for limited knowledge (such as guessing meanings from the context, etc.). | 英语老师教我们如何在现有知识不足的情况下解决问题（如通过上下文推测生词词意等）。 |
| TS02 The English teacher imparts practical knowledge to us (such as sentence patterns, etc.). | 英语老师教我们 “干货”知识（如写作句型等）。 |
| TS03 The English teacher pays careful attention to my studies. | 英语老师很关心我的学习情况。 |
| TS04 The English teacher has high expectations of me. | 英语老师对我有很高的期望。 |
| TS05 The English teacher helps me choose suitable learning materials. | 英语老师帮我选择合适的教辅资料。 |
| TS06 The English teacher helps me choose suitable extra-curricular reading materials. | 英语老师帮我选择合适的课外读物。 |
| PS01 My classmates would make me feel better when I have difficulties in learning English. | 当我的英语学习有困难的时候，我同学会帮助我。 |
| PS02 My classmates would pick me for a partner when dealing with English tasks (e.g. role playing, classroom activities). | 我同学会邀请我参加他/她的小组，一起完成英语学习任务（如角色扮演、课堂活动）。 |
| PS03 My classmates would share English learning resources with me. | 我同学会与我分享英语学习资源。 |
| PS04 My classmates would point out my mistakes in English learning and encourage me. | 我同学会指出我英语学习中的错误并鼓励我。 |
| FS01 At home, there is an adult who is interested in my English school work. | 家长对我的英语学业感兴趣。 |
| FS02 At home, there is an adult who wants me to do my best to learn English. | 家长希望我竭尽全力学习英语。 |
| FS03 My parents encourage me when I make progress in learning English. | 在我的英语学习取得进步的时候，家长对我说鼓励的话。 |
| FS04 My parents assist me in learning English. | 家长辅导我的英语学习。 |
| FS05 At home, there is an adult who believes that I will be a success in learning English. | 家长相信我的英语学习会取得成功。 |
| FS06 My parents made an effort to find a way to enroll me in the class I am currently attending. | 家长想办法把我送到现在就读的班级。 |
| FS07 My parents pay for my English tutoring classes. | 家长花钱给我补习英语。 |
| FS08 My parents set me an example with their own experiences in learning English. | 家长用自己的英语学习经历给我树立榜样。 |

*Note.* The items and their categories after CFA are as follows.

Positive individual characteristics (PC): PC01, PC02, PC03, PC04, PC05, PC06, PC07, PC12;

Teacher support (TS): TS01, TS02, TS04, TS05, TS06;

Peer support (PS): PS01, PS02, PS03, PS04;

Family support (FS): FS01, FS02, FS03, FS04.

1. The Liu’s (2013) study was initially published in Chinese and subsequently underwent revisions before being published in English in 2024. [↑](#footnote-ref-0)
